# Visuo-spatial (but not verbal) executive working memory capacity modulates susceptibility to non-numerical visual magnitudes during numerosity comparison

**DOI:** 10.1371/journal.pone.0214270

**Published:** 2019-03-27

**Authors:** Kyungmin Lee, Soohyun Cho

**Affiliations:** Department of Psychology, Chung-Ang University, Seoul, South Korea; University of Konstanz, GERMANY

## Abstract

The present study tested whether visuo-spatial vs. verbal executive working memory capacity (hereafter EWM) modulates the degree to which non-numerical visual magnitudes influence numerosity comparison using pairs of dot arrays. We hypothesized that visuo-spatial (rather than verbal) EWM capacity would influence one’s ability to selectively focus on numerical as opposed to non-numerical visual properties (such as dot size, cumulative area, density) of the dot arrays during numerosity comparison. Participants’ performance was better on trials in which non-numerical visual magnitudes were negatively (vs. positively) correlated with numerosity (i.e., reverse congruency effect). The Low visuo-spatial EWM group manifested greater reverse congruency effect compared to the High visuo-spatial EWM group. A trial-based hierarchical regression on the accuracy of each trial using the ratio of (numerical and non-numerical) visual magnitudes as predictors revealed that the ratio of numerical vs. non-numerical visual magnitudes explained the greatest variance in the performance of the High vs. Low visuo-spatial EWM groups, respectively. In contrast, there was no difference between the High vs. Low verbal EWM groups from the same analysis. These results reveal differential susceptibility to numerical vs. non-numerical visual information depending on the capacity of visuo-spatial (but not verbal) EWM. Taken together, numerosity comparison performance measured with the dot comparison paradigm seems to reflect not only one’s acuity for numerosity discrimination but also visuo-spatial EWM capacity likely required during integration of visual magnitudes during numerosity comparison.

## Introduction

Mathematical competence is an essential skill not only for academic achievement but also for everyday life [1, 2]. In the modern society where math and science have important roles, mathematical competence is critical not only to thrive at the individual level, but also for the competitiveness at the national level [3, 4]. The approximate number system has been proposed as the cognitive system that enables estimation of and approximate operation on discrete quantity representations (i.e. numerosity) [5–7]. These primitive mathematical abilities are commonly referred to as approximate number sense (hereafter ANS). ANS is believed to have evolved due to its survival value and has been reported to be also present in non-human animals, including primates [8, 9], and other mammals [10–12], birds [13, 14], frogs [15] and even fish [16, 17].

Following Weber’s law, the difficulty of numerosity comparison depends on the ratio between the numerical magnitudes being compared (i.e., the ratio effect). For example, a newborn baby can discriminate between two sets of numerosities when one is (at least) more than three times the other one (1:3 ratio) [18]. This ratio is reported to decrease with development becoming 1:2 by 6 months, 5:6 during preschool years, 7:8 in adolescence and 10:11 in young adulthood [7, 19, 20]. There are also significant individual differences in numerosity discrimination ability (hereafter ANS acuity) which is often measured as the minimum ratio of numerosities that one can reliably discriminate, which is termed the internal Weber fraction (*w’*) [20–22].

The numerosity comparison task requires the participant to determine the array with the larger (or smaller) numerosity within a pair of dot arrays. Recently, it has been found that numerosity comparison occurs through integration of not only numerical but also non-numerical visual properties of the dot array such as individual dot size, cumulative surface area, the smallest contour area (i.e., convex hull) and dot density (i.e., the cumulative surface area divided by convex hull) (note, individual dot size inherently covaries with cumulative surface area and dot density) [23–31]. Thus, numerosity comparison performance is often facilitated or interfered with (i.e., congruency effect) when these visual properties are positively or negatively correlated with numerosity. Numerosity comparison performance is commonly facilitated when convex hull is positively correlated with numerosity [24–28, 30]. However, when the influence of convex hull is controlled for (or minimized), performance is often facilitated when cumulative surface area (or dot size/density) is negatively correlated with numerosity (i.e., reverse congruency effect) [24, 28, 32]. This reverse congruency effect of cumulative surface area may reflect the tendency to perceive more dots when the array consists of smaller dots.

The congruency (or reverse congruency) effect is thought to reflect the need for executive working memory (hereafter EWM) in order to selectively attend to numerosity information and to suppress the influence of non-numerical visual properties of dot arrays during numerosity comparison [25, 30, 33–37]. This idea is supported by the study reporting that the EWM predicts children’s ANS measured by numerosity comparison, non-symbolic number line estimation tasks [38]. In several studies, children’s mathematical achievement was correlated with performance on only the incongruent but not congruent trials (when convex hull was not controlled for) [25, 33]. Furthermore, when inhibitory control was entered as a predictor in a regression analysis, children’s numerosity comparison performance was no longer a significant predictor of math achievement. Although the importance of inhibitory control especially in children’s numerosity comparison has been emphasized by previous studies [25, 27, 33], the possible contribution of visuo-spatial EWM capacity to numerosity comparison has not been as thoroughly examined. Recently, Szucs and colleagues (2013) reported that visuo-spatial EWM was a significant predictor of dyscalculic children’s math scores as well as inhibitory control ability [36]. The importance of visuo-spatial EWM in numerosity comparison ability of dyscalculic children was also reported by Bugden & Ansari (2016). Dyscalculic children showed impaired performance on only the incongruent but not congruent trials and their visuo-spatial EWM also predicted performance on only the incongruent trials [37].

In line with these studies, we hypothesize that adults’ numerosity comparison ability would also be influenced by EWM capacity especially in the visuo-spatial domain. Numerosity comparison using side-by-side dot arrays requires maintenance of visuo-spatial information, switching between two sets of dot arrays, in addition to selectively attending to numerosity while inhibiting non-numerical visual information during integration of visual information. We believe that this complex process likely depends on EWM especially in the visuo-spatial domain in addition to inhibition. We focused on EWM as an individual’s capacity to maintain and process multiple sources of information simultaneously. This definition is based on Daneman and Carpenter (1980)’s conception of WM capacity which led to the design of the original ‘complex span’ tasks [39]. Although most of the WM research in experimental psychology has focused on specifying the characteristics of Baddeley’s WM system [40], in psychometric psychology, there has also been a long-standing tradition of measuring the ‘processing capacity of WM’. For example, Daneman and Carpenter (1980) developed the ‘reading span’ task as a way of measuring an individual’s EWM capacity [39]. In this task, participants were instructed to read individual sentences and remember the word presented after each sentence for later recall. After a series of sentences, participants were prompted to recall those words in serial order. The logic of the reading span as a measure of WM capacity is that reading the sentences would prevent rehearsal of the to-be-remembered words and thus would require the participant to use EWM for maintenance and recall. Therefore, an individual who could only recall two words would have a smaller EWM capacity than an individual who was able to recall five words. EWM measured by complex span tasks such as the reading span task was found to be dissociable from inhibition ability [36, 37, 41–43].

As the main goal of the present study, we examined whether visuo-spatial (compared to verbal) EWM capacity is related to one’s ability to accurately and efficiently integrate visual information focusing on numerical (as opposed to non-numerical) information during numerosity comparison on a trial-by-trial basis. Given that this trial-by-trial analysis does not allow us to use individual EWM scores as a continuous variable, we divided our subjects into High vs. Low EWM (median split) groups (separately for visuo-spatial and verbal domains). We hypothesized that the High visuo-spatial (but not verbal) EWM group would be better at integrating visual information focusing on numerical (as opposed to non-numerical) information during numerosity comparison. As the behavioral task, we used a numerosity comparison task with a pair of dot arrays while holding convex hull constant across all arrays. We divided trials evenly into congruent and incongruent conditions in which individual dot size, cumulative surface area and dot density were all positively (congruent condition) vs. negatively (incongruent condition) correlated with numerosity. Based on previous reports, we expected to find better performance on incongruent trials (i.e., reverse congruency effect) given that convex hull was held constant. We hypothesized that this reverse congruency effect would be reduced in the High compared to Low visuo-spatial (but not verbal) EWM group.

As the main analysis of the present study, we tested whether visuo-spatial EWM modulates the susceptibility to non-numerical visual information during numerosity comparison on a trial-by-trial basis for the High vs. Low EWM groups, separately. We hypothesized that the Low visuo-spatial (but not verbal) EWM group would be more susceptible to non-numerical visual information during numerosity comparison compared to the High visuo-spatial EWM group. To test this hypothesis, we conducted a trial-based hierarchical regression analysis on the average performance of each trial using the ratio of visual magnitudes (numerosity and non-numerical visual magnitudes) on each trial as predictors. In the numerosity comparison task, the ratios of various visual magnitudes are known to influence performance [23–31]. Given that the ratio of each visual magnitude differs for each trial, the examination of the contribution of each visual magnitude on numerosity comparison performance needs to be conducted on a trial-by-trial basis. This analysis enables us to identify which visual magnitude(s) influence(s) performance and the degree to which it/they explain(s) the variance in performance on a trial-by-trial basis. Given that this trial-based approach analyzes how a certain trial is performed by a group of individuals, subject-based information cannot be analyzed at the same time. In other words, the trial-based analysis does not allow the use of EWM scores as a continuous, between-subject variable (see S1 Appendix and S1 Table). Hence, the trial-based analysis was conducted separately for High vs. Low EWM median split groups.

## Materials and methods

### Participants

Forty-two undergraduate students aged between 19 and 25 years (mean = 20.8, SD = 1.9; 33 females) participated in the study. All participants had normal or corrected-to-normal vision without a history of neurological or psychiatric disorders. The Institutional Review Board (IRB) of Chung-Ang University approved all protocols of the study (1041078-201312-HR-0105-02). Written informed consent was acquired from each participant prior to the experiment.

### Tasks and materials

#### Numerosity comparison task

Two arrays of dots appeared side by side simultaneously on the left and right side of the screen for 1000 ms. On each trial, participants were instructed indicate their choice of the array with the larger numerosity by pressing the 3(left) or 8(right) keys on the keyboard as fast and as accurately as possible.

**Fig 1:**
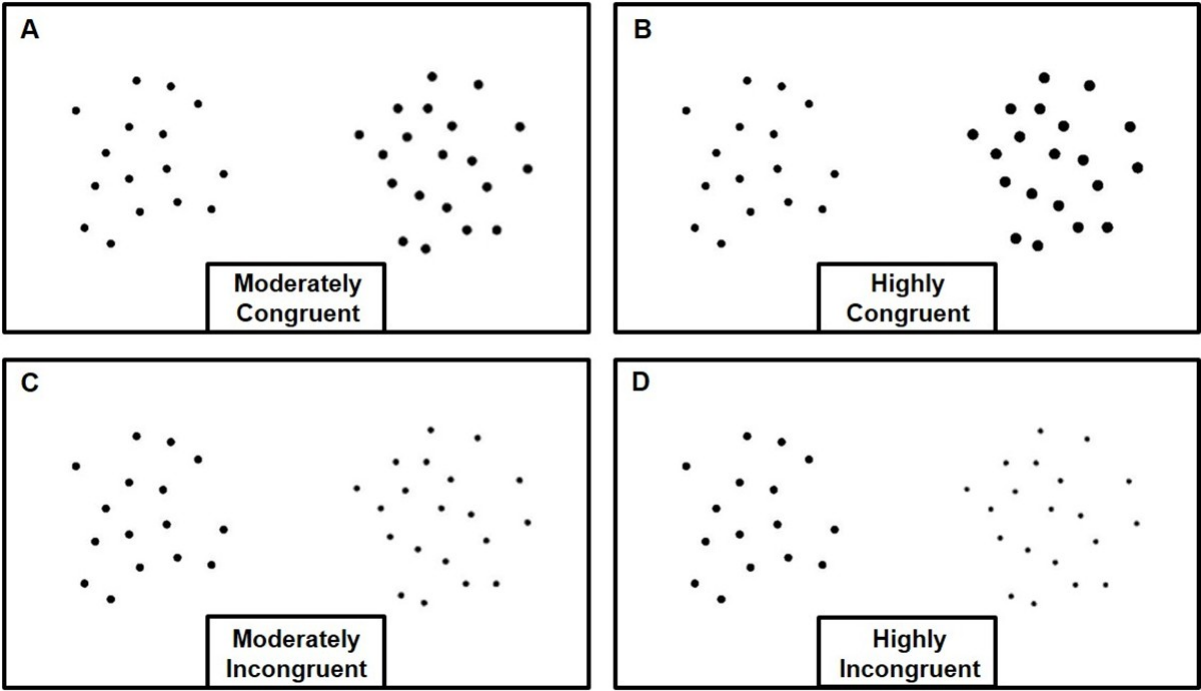
Example stimuli (16 vs. 20) of each condition of the Numerosity Comparison task.

**Fig 2:**
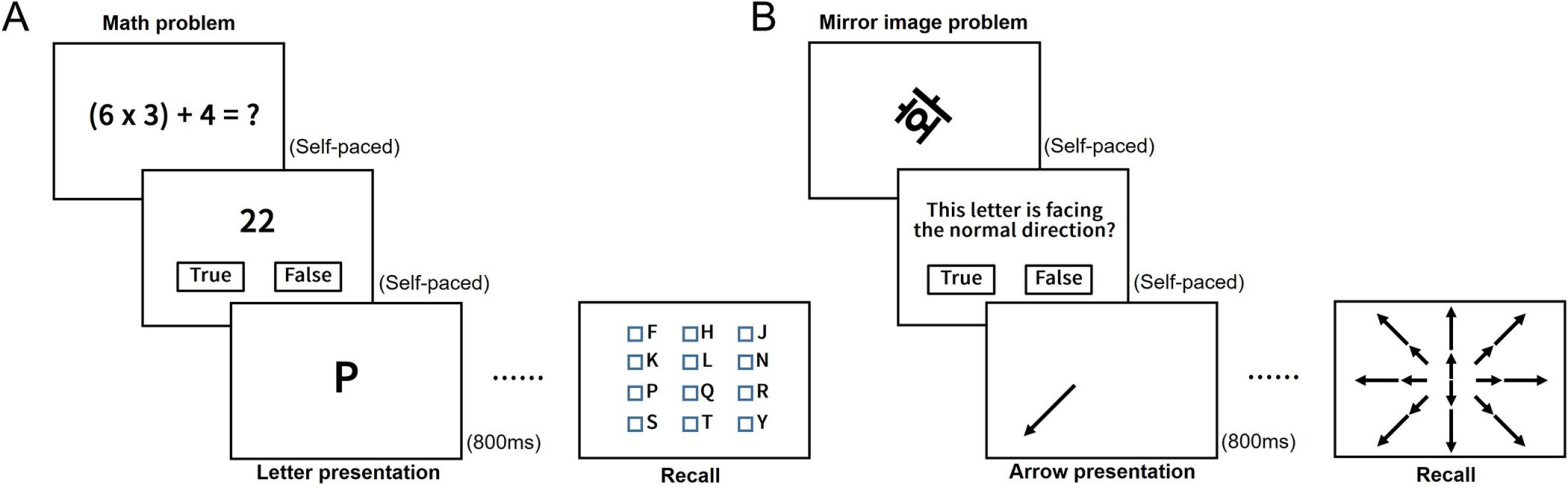
**Task procedure for the (A) Operation and (B) Rotation Span tasks [44]**.

**Fig 3:**
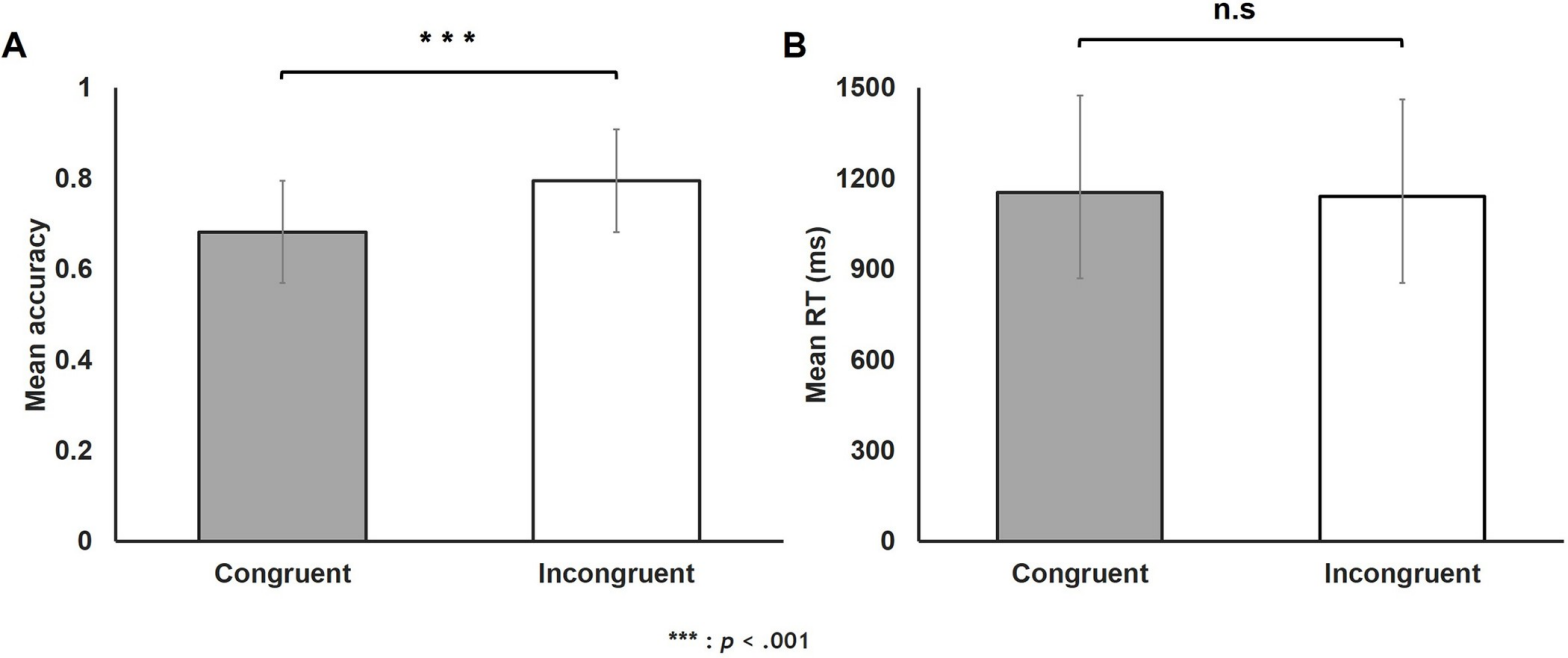
**Results of dependent samples *t*-tests on mean (A) accuracy and (B) RT from the Numerosity Comparison task.** Accuracy (but not RT) was significantly higher in the Incongruent compared to the Congruent condition manifesting a reverse congruency effect. Error bars represent standard errors of the mean.

**Fig 4:**
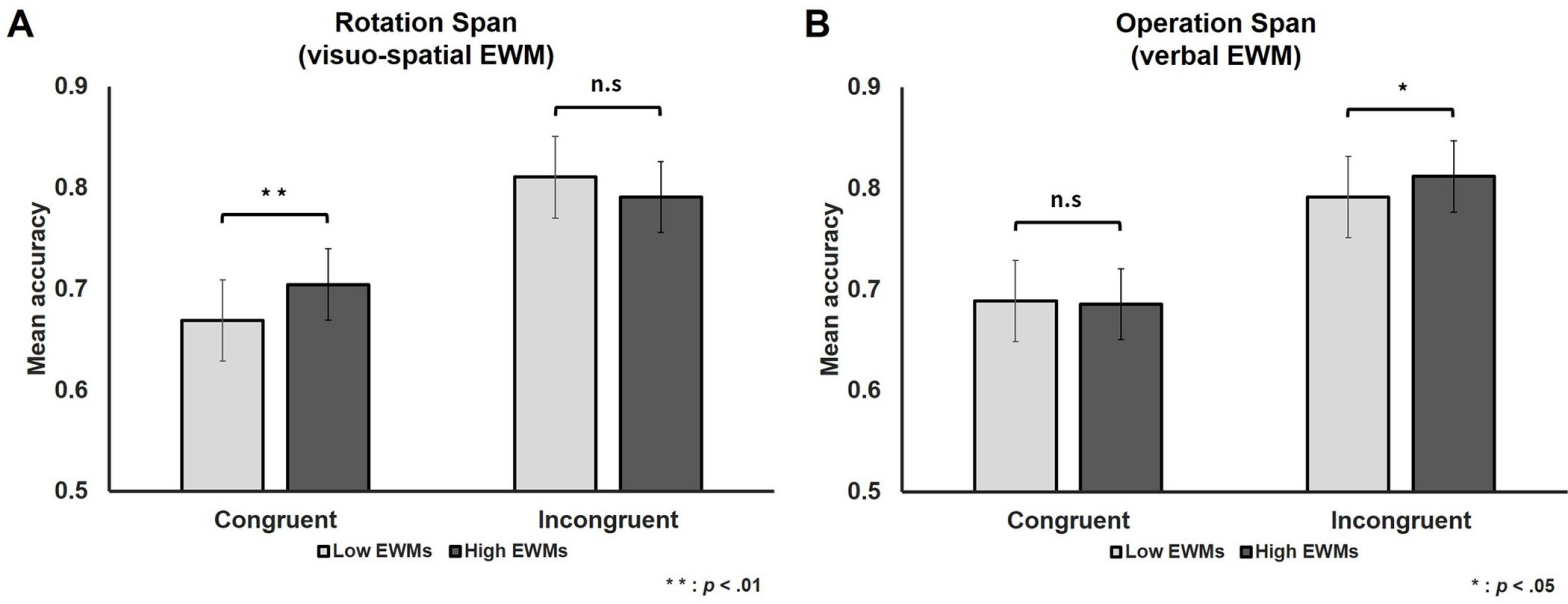
**Results of ANCOVA on Numerosity Comparison accuracy using (A) visuo-spatial and (B) verbal EWM groups as within-trial factors and Congruency as between-trial factors** (along with IDS ratio and RT as covariates of non-interest). There was a significant two-way interaction between Congruency and visuo-spatial EWM Group (A). There was a no significant two-way interaction between Congruency and verbal EWM Group (B). Error bars represent standard errors of the mean.

Dot array stimuli were generated with variation in visual properties such as individual dot size, cumulative surface area and dot density. Convex hull size, which is reported to have a greater impact on performance than other visual properties, was held constant across all stimuli [28, 30]. On each trial, the ratios of visual magnitudes between each pair of dot arrays were calculated. Congruent conditions refer to the trials in which numerosity was positively correlated with non-numerical visual magnitudes (including dot size, cumulative area and density which are all inherently related to one another). In contrast, Incongruent conditions refer to the trials in which numerosity was negatively correlated with non-numerical visual magnitudes (including dot size, cumulative area and density). Trials were divided into four conditions based on the degree of (in)congruency; 1) Moderately Congruent (MC): non-numerical visual magnitudes were positively correlated with numerosity and the ratio of visual magnitudes were in the range of 1.07–1.43 (Fig 1A); 2) Highly Congruent (HC): non-numerical visual magnitudes were highly positively correlated with numerosity and the ratio of visual magnitudes were in the range of 1.29–1.71 (Fig 1B); 3) Moderately Incongruent (MI): non-numerical visual magnitudes were negatively correlated with numerosity and the ratio of visual magnitudes were in the range of 0.70–0.93 (Fig 1C); 4) Highly Incongruent (HI): non-numerical visual magnitudes were highly negatively correlated with numerosity and the ratio of visual magnitudes were in the range of 0.58–0.78 (Fig 1D). The ratio of inter dot space (hereafter IDS) which refers to the average distance between the center of all possible pairs of dots within an array could not be systematically controlled, thus was calculated on each trial and was used as a covariate in statistical analyses. Detailed ranges of visual magnitudes are provided in the supporting information (S1 Appendix, S1 Fig and S2 Table).

The set size of dots varied from 12 to 40. The difficulty of numerosity comparison was determined by the numerosity ratio between the dot arrays within each pair which varied from 3:4 to 8:9 (3:4, 4:5, 5:6, 6:7, 7:8, 8:9). There were 40 trials for each ratio, adding up to a total number of 240 trials per participant. The order of presentation was randomly intermixed across ratios and conditions. Three practice trials with feedback were administered before the main experiment.

#### Operation span task (Verbal EWM)

We used the Operation Span task to measure verbal EWM [44]. Participants were required to remember a sequence of letters of the alphabet (main task) and also to solve a math problem (subtask). On each trial, a math problem (e.g., (5 x 4) + 3 = ?) was presented. When the participant pressed the space bar (self-paced), a possible answer (e.g., 23) was shown. Participants responded by clicking on one of two square boxes labeled “True” or “False” on the monitor (self-paced). Then, a letter (e.g., “K”) was presented on the screen for 800ms and the participant was asked to remember it. The next trial began immediately after the letter disappeared. The math problem and letter pair was repeated three to seven times (i.e., span size) per trial. At the end of each trial, the participant was asked to recall the sequence of letters in order by clicking on appropriate cells of a 4 × 3 matrix which contained the target and distractor letters. The span size tested ranged from 3–7. For each participant, each span size was repeated twice for a total of 10 trials in random order. Participants were encouraged to do their best on both the main and subtasks. Fifteen practice trials with feedback were administered before the main experiment. The entire procedure of the operation span task is shown in Fig 2A.

#### Rotation span task (Visuo-spatial EWM)

We used the Rotation Span task to measure visuo-spatial EWM [44] with a small modification. Participants were asked to remember a sequence of arrows with two different lengths and eight different directions (main task) and also to solve a problem (subtask) of judging whether a Korean syllable (e.g., “화”) was presented as a mirror image or not (In contrast to Foster and colleagues in which a letter of the alphabet was used [44], the present study used a Korean syllable because all participants were native Korean speakers). The syllables used were “월”, “화”, “목”, “금” which corresponds to Monday, Tuesday, Thursday and Friday, respectively. A pair of arrow and distractor problem was repeated from two to five times (span size) per trial. At the end of each trial, the participant was asked to recall the sequence of arrows by clicking on the correct images of arrows among 16 options. The span size tested ranged from 2–5. For each participant, each span size was repeated twice for a total of 8 trials in random order. The experimental procedure including the number of practice trials was otherwise identical to that of the operation span task. The entire procedure of the rotation span task is shown in Fig 2B. Additional information about the operation and rotation span tasks are also provided in the supporting information (S1 Appendix).

### Analyses

For quality control, correct trials with RTs beyond ± 2.5 SDs from the mean of each individual were classified as incorrect in the numerosity comparison task (which amounted to about 2% of the total number of correct trials). Participants with performance scores beyond ± 3 SDs from the group mean of each task were meant to be excluded from data analysis but none of the participants met this criterion, thus all participants’ data were included in the analysis. The EWM score for each individual was calculated according to the scoring method by Unsworth et al. (2005) and Redick et al. (2012). This method adds up the span from trials in which all items were recalled in the correct order. For example, if the participant correctly recalled all three items of the first trial (Span 3), three among the four items of the second trial (Span 4), and all five items of the third trial (Span 5), then the participant’s score would be 8 (3+0+5) [45, 46]. Possible scores ranged from 0–50 in the operation span and 0–28 in the rotation span task. Participants were classified into High vs. Low EWM groups based on a median split of each EWM score resulting in High vs. Low verbal and visuo-spatial EWM groups [47].

Two different methods of statistical analysis were conducted on numerosity comparison task performance (mean accuracy and RT): 1) a subject-based analysis in which task performance of all participants were used as dependent variables, 2) a trial-based analysis in which the group average of performance on each trial were used as the dependent variable for each group. For these analyses, performance on combined Congruent (HC+MC) conditions was contrasted with performance on combined Incongruent (HI+MI) conditions. The subject-based analysis was conducted to confirm the presence of a reverse congruency effect across all participants. The trial-based analysis was conducted by an ANCOVA and regression analysis on the group average of performance on each trial using the ratio of visual magnitudes (numerosity and non-numerical visual magnitudes) as covariates (in the ANCOVA) or predictors (in the regression analysis) for each group separately (note, in this trial-based analysis, it is not possible to use EWM scores as a continuous, between-subject variable; see S1, S3 and S4 Tables).

## Results

### Behavioral performance

The mean scores of the Operation Span (verbal EWM) and Rotation Span (visuo-spatial EWM) were 29.83 (SD = 10.68) and 16.74 (SD = 4.95), respectively (see S2 Fig for the distribution of each EWM score). The correlation between the verbal and visuo-spatial spans was not significant (*r* = .256, *p* = .10). There were 21 individuals in the high and low EWM groups based on a median split. The mean scores of the Operation Span of the High and Low verbal EWM groups were 38.38 (SD = 6.52) and 21.29 (SD = 6.13), respectively and this difference was significant (*t* (40) = 8.75, *p* < .001). The mean scores of the Rotation Span of the High and Low visuo-spatial EWM were 20.62 (SD = 3.15) and 12.86 (SD = 2.94), respectively and this difference was significant (*t* (40) = 8.25, *p* < .001).

The means and ranges of performance on the Numerosity Comparison task are shown in Table 1. Mean performance of each group on the Congruent (Moderately Congruent, Highly Congruent) and Incongruent (Moderately Incongruent, Highly Incongruent) conditions are shown in Table 2.

**Table 1:** The means and ranges of Numerosity Comparison performance (accuracy, RT) in each condition.

	Moderately Congruent	Highly Congruent	Moderately Incongruent	Highly Incongruent	TOTAL
**Mean accuracy**	0.77 (0.09)	0.62 (0.15)	0.82 (0.10)	0.81 (0.13)	0.75 (0.06)
**Range**	0.50–0.95	0.25–0.88	0.52–1	0.47–1	0.59–0.84
**Mean RT (ms)**	1152 (301)	1164 (371)	1132 (287)	1146 (278)	1142 (291)
**Range (ms)**	541–1879	530–2620	573–1615	600–1565	560–1773

**Table 2:** Numerosity Comparison performance (accuracy, RT) of each group in congruent vs. incongruent trials.

		High verbal EWM	Low verbal EWM	High visuo-spatial EWM	Low visuo-spatial EWM
**Accuracy**	**Congruent**	0.67 (0.10)	0.69 (0.12)	0.70 (0.11)	0.67 (0.11)
	**Incongruent**	0.81 (0.13)	0.78 (0.09)	0.79 (0.12)	0.80 (0.11)
	**Total**	0.74 (0.05)	0.73 (0.06)	0.74 (0.05)	0.74 (0.06)
**RT (ms)**	**Congruent**	1232 (332)	1077 (295)	1194 (264)	1115 (370)
	**Incongruent**	1212 (255)	1065 (295)	1178 (254)	1100 (309)
	**Total**	1215 (275)	1069 (295)	1183 (256)	1101 (324)

### The reverse congruency effect

Dependent-samples *t*-test on accuracy from the Numerosity Comparison task revealed a significant difference between Congruent vs. Incongruent conditions (*t* (41) = 3.80, *p* < .001; Fig 3A). Accuracy was significantly higher in the Incongruent compared to the Congruent condition manifesting a reverse congruency effect. However, the same analysis on RT revealed no significant difference between conditions (*t* (41) = 0.86, *p* = .40; Fig 3B). A correlation analysis between accuracy and RT revealed a speed-accuracy trade off (*r* = .65, *p* < .001). Thus, mean RT (or accuracy) was used as a covariate in subsequent analyses on accuracy (or RT).

### Between-group difference in reverse congruency effects

We conducted a 2 x 2 (Group x Congruency) mixed repeated measures ANCOVA on trial-by-trial accuracy using Group as a within-trial factor, Congruency as a between-trial factor, along with IDS ratio and RT as covariates of non-interest. Analysis on accuracy using visuo-spatial EWM Group (High vs. Low) as a within-trial factor revealed a significant two-way interaction between Congruency and Group (*F*(1, 236) = 12.43, *p* = .001). Post-hoc t tests revealed that the between group difference was significant in the Congruent (*t*(119) = 3.13, *p* = .002), but not Incongruent (*t*(119) = 1.68, *p* = .10) condition. The reverse congruency effect was greater in the Low compared to High visuo-spatial EWM group (Fig 4). Furthermore, the two-way interaction remained significant even when the numerosity ratio of each trial was used as a covariate (*F*(1, 235) = 12.70, *p* < .001). This result implies that the numerosity ratio did not influence the two-way interaction between Congruency and Group. In contrast, when the dot size ratio was used as an additional covariate, the two-way interaction was no longer significant (*F*(1, 235) < 0.001, *p* = .99). (Note, the ratio of cumulative area and density was not additionally used here to avoid problems related to multicollinearity). The same ANCOVA on trial-by-trial RT showed no significant two-way interaction effect (*F*(1, 235) = 1.06, *p* = .30).

The same ANCOVA on trial-by-trial accuracy or RT using verbal EWM Group (High vs. Low) as a within-trial factor, Congruency as a between-trial factor revealed no significant two-way interaction effects (accuracy: *F*(1, 235) = 2.21, *p* = .14; RT: *F*(1, 235) = 3.48, *p* = .06).

### The influence of non-numerical visual magnitudes on trial-by-trial numerosity comparison performance

Given that the between group difference in Congruency effects were not significant from analyses of RT, subsequent analyses were only conducted on accuracy (RT was used as a covariate to regress out the influence of speed-accuracy trade-off). Hierarchical linear regression analyses were conducted to examine the influence of visual magnitude ratios on numerosity comparison performance for each Group. In all hierarchical regression analyses, we used the ratios of numerosity, dot size (note, the ratio of cumulative area and density was not used to avoid problems related to multicollinearity) and IDS (note, IDS ratio was not correlated with the ratios of other non-numerical visual magnitudes) as predictors and trial-by-trial accuracy as the dependent variable, separately for each group.

First, for both High and Low visuo-spatial EWM groups, all predictors explained unique variance in trial-by-trial accuracy in the final model (High: *F*(4, 235) = 44.64, *p* < .001, *R*^*2*^(3, 235) = 0.43; Low: *F*(4, 235) = 68.34, *p* < .001, *R*^*2*^(3, 235) = 0.54; Table 3). In both groups, the relationship between the numerosity ratio and performance was positive, reflecting better performance on trials with higher numerosity ratios, while the relationship between the size ratio and performance was negative, reflecting better performance on trials with lower size ratios (i.e., incongruent trials). In the High visuo-spatial EWM group, numerosity ratio explained the greatest variance in trial-by-trial accuracy, followed by the size ratio and then by IDS ratio (Table 3). In contrast, in the Low visuo-spatial EWM group, the size ratio explained the greatest variance in trial-by-trial accuracy, followed by numerosity ratio and then by IDS ratio (Table 3).

**Table 3:** Results of hierarchical regression on Numerosity Comparison accuracy of high vs. low visuo-spatial EWM groups.

Steps	Predictor	High visuo-spatial EWM group			Low visuo-spatial EWM group		
		*β*	*sr*^*2*^	*ΔR*^*2*^	*β*	*sr*^*2*^	*ΔR*^*2*^
**Step 1**	**RT**	- 0.347***	0.121	0.121	- 0.383***	0.147	0.147
**Step 2**	**RT**	- 0.182**	0.029	0.311	- 0.292***	0.078	0.391
	**Numerosity ratio**	0.477^***^	**0.176**		0.392***	0.126	
	**Size ratio**	- 0.322***	0.102		- 0.477^***^	**0.225**	
	**IDS ratio**	0.251***	0.055		0.205***	0.036	

N = 240. *β*: standardized regression coefficient; *sr*^*2*^: squared semi-partial correlation

^**^p < 0.01

^***^p < 0.001

Secondly, for both High and Low verbal EWM groups, all predictors explained unique variance in trial-by-trial accuracy in the final model (High: *F*(4, 235) = 47.91, *p* < .001, *R*^*2*^(3, 235) = 0.45; Low: *F*(4, 235) = 60.11, *p* < .001, *R*^*2*^(3, 235) = 0.51; Table 4). In both groups, the relationship between the numerosity ratio and performance was positive, reflecting better performance on trials with higher numerosity ratios, while the relationship between the size ratio and performance was negative, reflecting better performance on trials with lower size ratios (i.e., incongruent trials). In both High and Low verbal EWM groups, the numerosity ratio explained the greatest variance in trial-by-trial accuracy, followed by size ratio and then by IDS ratio.

**Table 4:** Results of hierarchical regression on Numerosity Comparison accuracy of high vs. low verbal EWM groups.

Steps	Predictor	High verbal EWM group			Low verbal EWM group		
		*β*	*sr*^*2*^	*ΔR*^*2*^	*β*	*sr*^*2*^	*ΔR*^*2*^
**Step 1**	**RT**	- 0.051	0.003	0.003	- 0.495***	0.245	0.245
**Step 2**	**RT**	- 0.054	0.003	0.447	- 0.287***	0.070	0.261
	**Numerosity ratio**	0.515^***^	**0.225**		0.437^***^	**0.151**	
	**Size ratio**	- 0.415***	0.162		- 0.316***	0.096	
	**IDS ratio**	0.236***	0.049		0.259***	0.057	

N = 240. *β*: standardized regression coefficient; *sr*^*2*^: squared semi-partial correlation

^***^p < 0.001

## Discussion

The present study tested whether group differences in visuo-spatial vs. verbal EWM impacts numerosity comparison performance and the susceptibility to numerical vs. non-numerical visual information during task performance. Overall, the participants’ performance was better on incongruent compared to congruent trials (i.e., reverse congruency effect). The Low visuo-spatial EWM group manifested a greater reverse congruency effect compared to the High visuo-spatial EWM group. Trial-based hierarchical regression analyses revealed that the numerosity ratio explained the greatest variance in the performance of the High visuo-spatial EWM group. In contrast, the size ratio explained the greatest variance in the performance of the Low visuo-spatial EWM group. There were no differences between the Low vs. High verbal EWM groups.

### The reverse congruency effect in numerosity comparison performance

In the present study, mean accuracy on incongruent trials was significantly higher than that of congruent trials of the numerosity comparison task. This reverse congruency effect is consistent with the results of Gebuis and Reynvoet [24] and Clayton and colleagues [28]. In Clayton and colleagues [28], a congruency effect or a reverse congruency effect was observed depending on whether or not convex hull size was informative for task performance. When convex hull size was positively correlated with numerosity, a congruency effect was observed. In contrast, when there was small variation in convex hull size, participants were influenced by dot size/cumulative area rather than convex hull, leading to better performance when dot size/cumulative area were negatively correlated with numerosity [28]. Similarly, in Gebuis and Reynvoet [24] when convex hull size was kept constant, performance was better on trials in which non-numerical visual magnitude was negatively correlated with numerosity. Taken together, these results suggest that when convex hull size information is not informative, participants tend to be influenced by non-numerical visual magnitudes such as dot size (i.e., participants tend to perceive more dots when the array consists of smaller compared to bigger dots). Gebuis and Reynvoet [24] proposed that participants integrate information from multiple visual cues including both numerical and non-numerical cues during numerosity comparison [23, 24]. The results of our trial-by-trial analysis support their interpretation by demonstrating that the ratio of numerosities and non-numerical visual magnitudes both influence performance. It seems possible that a congruency or reverse congruency effect can be observed depending on the strength of the influences from multiple sources of information during the integration process.

### The low visuo-spatial EWM group shows a larger reverse congruency effect and greater susceptibility to non-numerical visual magnitudes

In the present study, we classified participants into Low vs. High EWM groups and compared the degree of congruency effects between groups using ANCOVA. The Low visuo-spatial EWM group manifested a greater reverse congruency effect compared to the High visuo-spatial EWM group (Fig 4A). The Low visuo-spatial EWM group showed a significantly lower accuracy on the congruent compared to incongruent trials. In addition, these results remained the same even when the numerosity ratio was added as a covariate, indicating that these results were not influenced by how numerosity information was used for numerosity comparison. On the other hand, when the size ratio was added as a covariate, the performance difference between groups did not remain significant, indicating that these results were influenced by the between-group difference in susceptibility to non-numerical visual information. Interestingly, there was no significant difference between Low vs. High verbal EWM groups, suggesting that the congruency effect is related to visuo-spatial, rather than verbal EWM capacity (Fig 4B). The present observation of a greater reverse congruency effect in the Low visuo-spatial EWM group suggests the possibility that visuo-spatial EWM capacity impacts the ability to focus on numerosity and disregard non-numerical visual magnitude information during numerosity comparison.

In addition, hierarchical regression analyses revealed that the numerosity ratio explained the greatest variance in the performance of the High visuo-spatial EWM group, while the size ratio explained the greatest variance in the performance of the Low visuo-spatial EWM group. These results reveal differential susceptibility to numerical vs. non-numerical visual information depending on the capacity of visuo-spatial EWM.

Taken together, the present study demonstrated that visuo-spatial EWM modulates the ability to focus on numerosity and to disregard non-numerical visual magnitude information, thereby influences the pattern and degree of the (reverse) congruency effect during numerosity comparison.

### Implications for developmental studies of ANS and its relationship to mathematical achievement

While some studies report positive correlations between ANS acuity and high level mathematical achievement, other studies failed to find evidence for this relationship [48–56]. Part of the cause of this discrepancy may be attributed to methodological differences across studies especially in relation to the design of the numerosity comparison task [30, 57, 58]. Given that non-numerical visual information impacts numerosity comparison performance, how the visual properties of the stimuli are designed may greatly influence the demand for EWM resources during task performance. The differential demand on domain-general ability such as EWM may lead to different combination of abilities being measured by the numerosity comparison task [25, 30, 33].

Although the present study was conducted on young adults and therefore what was found here may not be exactly replicated in children or the aging population, we carefully speculate that the requirement for visuo-spatial EWM during numerosity comparison is likely to be generalizable across different age groups. The fact that numerosity comparison demands visuo-spatial EWM may help explain reported differences in numerosity comparison performance across age groups. For example, reports of a greater congruency effect in younger compared to older children may reflect improved EWM with development during childhood [30, 59]. Studies of children with developmental dyscalculia (DD) showed significantly impaired visuo-spatial EWM capacity and inhibitory control ability. These studies demonstrated that visuo-spatial EWM as well as inhibition plays an important role in numerosity comparison [36, 37]. Interestingly, children with DD demonstrated impaired performance only on incongruent trials in which inhibition was required to suppress the influence of non-numerical visual magnitudes. Bugden & Ansari (2013) speculated that impairment of visuo-spatial EWM or inhibitory control (or both) may lead to greater susceptibility to non-numerical visual information which ultimately hinders efficient processing of numerical information on incongruent trials [37]. Taken together with the result of the present study, younger children or individuals with low capacity for visuo-spatial EWM may show a greater (reverse) congruency effect because their task performance is more influenced by non-numerical visual information of the stimuli. In a similar vein, reports of the decline of numerosity comparison performance in older adults on incongruent trials may reflect the deterioration of visuo-spatial EWM [60, 61], in addition to the ANS and inhibition ability [34, 35].

### Limitations and suggestions for future research

Numerosity comparison ability is believed to be an important aspect of ANS. Recent studies reported that the dot comparison task commonly used to measure numerosity comparison ability is found to require not only ANS but also visuo-spatial EWM and inhibition ability, especially for trials in which numerosity is correlated with non-numerical visual characteristics of the dot arrays [25, 33, 36, 37]. This is reflected in the (reverse) congruency effects which were found to be aggravated in children with DD or individuals with low capacity for visuo-spatial EWM [36, 37]. Many researchers also emphasize that the ability to suppress the influence of non-numerical visual magnitudes during numerosity comparison critically requires inhibition ability [25, 27, 33, 36, 37]. Thus, in order to thoroughly examine the underlying mechanisms of numerosity comparison, future studies should include measures of both inhibition and visuo-spatial EWM along with other possible candidate mechanisms. In this respect, we acknowledge the lack of separate measurement of inhibition ability as a limitation of the present study. In order to track developmental changes in the contribution of inhibitory control and visuo-spatial EWM to numerosity comparison ability across the lifespan, longitudinal studies should be conducted on participants across a diverse age range.

## Conclusions

The present study demonstrated that visuo-spatial EWM capacity relates to the degree to which numerical vs. non-numerical visual magnitudes influence numerosity comparison performance. Taken together with previous studies, it seems clear that the numerosity comparison task is not a pure measure of ANS but rather a combination of abilities including domain general cognitive abilities such as visuo-spatial EWM and inhibitory control. Accurately accounting for the influence of non-numerical visual information on numerosity processing is crucial for the improvement of ANS measurement. Furthermore, understanding how numerical vs. non-numerical information is integrated during numerosity comparison will also be a way to test and develop the ‘sense of magnitude’ theory, which states that numerosities are not processed independently of non-numerical information, i.e., numerosities and continuous magnitudes are processed holistically due to the inherent correlation between numerosity and non-numerical information [62].

## Supporting information

S1 Table Example of the input data used for the trial-based analysis. (DOCX)

S2 Table The ratios of numerical and non-numerical visual magnitudes in each condition. (DOCX)

S3 Table The list of trial-based ANCOVAs on performance using Group as the within-trial factor, Condition as the between-trial factor along with covariates. (DOCX)

S4 Table The list of trial-based hierarchical regression analyses conducted on accuracy for each group. (DOCX)

S1 Fig The ratios of numerical and non-numerical visual magnitudes used in the stimuli of the numerosity comparison task. Trial types are depicted in four different ways, in terms of the relationships between the numerosity ratio and each non-numerical visual magnitude ratio. Each point represents a certain type of trial (contains 10 trials each). The horizontal line in each figure defines the boundary that separates the Congruent (> 1) and Incongruent (< 1) conditions.
(TIF)

S2 Fig The distributions of Verbal and Visuo-spatial EWM scores. The analysis of skewness and kurtosis revealed that none of the scores exceeded + or – 1.00.
(TIF)

S1 Appendix Supplementary methods and results. Here we provide additional descriptions about experimental tasks, analysis methods and behavioral performance on operation and rotation span tasks.
(DOCX)
