# Supplementary material for: Visuo-spatial (but not verbal) executive working memory capacity modulates susceptibility to non-numerical visual magnitudes during numerosity comparison
Source: PLoS One. 2019 Mar 27;14(3):e0214270. doi: 10.1371/journal.pone.0214270 (PMC6436736; doi:10.1371/journal.pone.0214270)
Supplement: S1 Table — (DOCX) [file pone.0214270.s001.docx]

**S1 Table. Example of the input data used for the trial-based analysis.**

| **Trial ID** | **Numeorsity ratio** | **Size ratio** | **Area ratio** | **Density ratio** | **IDS ratio** | **Condition** | **High verbal EWM ACC** | **High verbal EWM RT** | **Low verbal EWM ACC** | **Low verbal EWM RT** | **High visuo EWM ACC** | **High visuo EWM RT** | **Low visuo EWM ACC** | **Low visuo EWM RT** |
| --- | --- | --- | --- | --- | --- | --- | --- | --- | --- | --- | --- | --- | --- | --- |
| 1 | 1.33 | 1.29 | 1.71 | 1.71 | 0.85 | HC | 0.9 | 1085.84 | 1 | 947.14 | 0.95 | 1041.55 | 0.95 | 984.5 |
| 2 | 1.33 | 1.07 | 1.43 | 1.43 | 0.83 | MC | 0.95 | 1163.95 | 0.86 | 1051.94 | 0.95 | 1102.4 | 0.86 | 1120.33 |
| 3 | 1.33 | 0.70 | 0.93 | 0.93 | 0.86 | MI | 0.9 | 1181.68 | 0.9 | 1002.26 | 0.95 | 1060.3 | 0.81 | 1058.88 |
| 4 | 1.33 | 0.58 | 0.78 | 0.78 | 0.87 | HI | 1 | 1277.24 | 0.86 | 1114.33 | 0.95 | 1252.9 | 0.9 | 1148.53 |
| 5 | 1.25 | 1.33 | 1.67 | 1.67 | 0.83 | HC | 0.86 | 973.33 | 0.76 | 963.88 | 0.86 | 936.11 | 0.76 | 1005.75 |
| 6 | 1.25 | 1.11 | 1.39 | 1.39 | 0.88 | MC | 0.81 | 1128.24 | 0.76 | 1193.5 | 0.81 | 1192.65 | 0.81 | 1216.76 |
| 7 | 1.25 | 0.72 | 0.90 | 0.90 | 1.01 | MI | 1 | 1198.9 | 0.95 | 1085 | 1 | 1186.29 | 0.95 | 1098.25 |
| 8 | 1.25 | 0.60 | 0.75 | 0.75 | 0.88 | HI | 0.9 | 1302.79 | 0.95 | 1068.65 | 0.95 | 1251.8 | 0.9 | 1110 |
| 9 | 1.23 | 1.34 | 1.66 | 1.66 | 0.97 | HC | 0.62 | 1301.85 | 0.71 | 1152.87 | 0.71 | 1244.33 | 0.62 | 1196.31 |
| 10 | 1.23 | 1.12 | 1.38 | 1.38 | 1.01 | MC | 0.67 | 1300.71 | 0.48 | 997.5 | 0.62 | 1259.54 | 0.52 | 1073.73 |
| 11 | 1.23 | 0.72 | 0.89 | 0.89 | 0.91 | MI | 0.86 | 1246.39 | 0.86 | 1072.72 | 0.86 | 1095.78 | 0.81 | 1142.82 |
| 12 | 1.23 | 0.60 | 0.74 | 0.74 | 0.97 | HI | 0.95 | 1300 | 0.81 | 1118.71 | 0.9 | 1260.21 | 0.86 | 1170.78 |
| 13 | 1.19 | 1.37 | 1.63 | 1.63 | 0.92 | HC | 0.39 | 1080.5 | 0.63 | 1031.33 | 0.58 | 1240.38 | 0.44 | 736.2 |
| 14 | 1.19 | 1.14 | 1.36 | 1.36 | 0.85 | MC | 0.71 | 1250.8 | 0.67 | 1017.93 | 0.71 | 1240.27 | 0.67 | 1029.21 |
| 15 | 1.19 | 0.74 | 0.87 | 0.87 | 0.90 | MI | 0.71 | 1337.53 | 0.67 | 1303.36 | 0.81 | 1295.12 | 0.57 | 1357.75 |
| 16 | 1.19 | 0.61 | 0.73 | 0.73 | 0.88 | HI | 0.86 | 1209.06 | 0.71 | 1010.13 | 0.76 | 1146.06 | 0.81 | 1092.82 |
| 17 | 1.14 | 1.4 | 1.6 | 1.6 | 1.02 | HC | 0.43 | 1321.11 | 0.52 | 1100.64 | 0.52 | 1220.45 | 0.43 | 1174.67 |
| 18 | 1.14 | 1.17 | 1.33 | 1.33 | 0.95 | MC | 0.52 | 1346.09 | 0.71 | 1202.67 | 0.67 | 1225.79 | 0.52 | 1231.09 |
| 19 | 1.14 | 0.75 | 0.86 | 0.86 | 1.06 | MI | 0.9 | 1227.21 | 0.95 | 1030.9 | 0.9 | 1218.26 | 0.95 | 1039.4 |
| 20 | 1.14 | 0.63 | 0.71 | 0.71 | 0.97 | HI | 0.95 | 1212.5 | 0.62 | 1080.54 | 0.71 | 1176.6 | 0.86 | 1147.11 |
| 21 | 1.13 | 1.41 | 1.59 | 1.59 | 0.95 | HC | 0.52 | 1122.45 | 0.52 | 989.73 | 0.62 | 1245.85 | 0.48 | 939.8 |
| 22 | 1.13 | 1.18 | 1.32 | 1.32 | 0.87 | MC | 0.62 | 1245.92 | 0.62 | 1314.77 | 0.62 | 1360.92 | 0.62 | 1199.77 |
| 23 | 1.13 | 0.76 | 0.85 | 0.85 | 0.94 | MI | 0.67 | 1281.64 | 0.62 | 1173.69 | 0.71 | 1185.8 | 0.57 | 1284.5 |
| 24 | 1.13 | 0.63 | 0.71 | 0.71 | 0.90 | HI | 0.86 | 1231.44 | 0.48 | 1197.6 | 0.67 | 1228.36 | 0.67 | 1210.36 |
