# Supplementary material for: Visuo-spatial (but not verbal) executive working memory capacity modulates susceptibility to non-numerical visual magnitudes during numerosity comparison
Source: PLoS One. 2019 Mar 27;14(3):e0214270. doi: 10.1371/journal.pone.0214270 (PMC6436736; doi:10.1371/journal.pone.0214270)
Supplement: S2 Table — (DOCX) [file pone.0214270.s002.docx]

**S2 Table. The ratios of numerical and non-numerical visual magnitudes in each condition.**

|  | **Numerosity ratio** | **Size ratio** | **Area ratio** | **Density ratio** | **Inter-dot space ratio** |
| --- | --- | --- | --- | --- | --- |
| Moderately congruent (MC) | 1.13 – 1.33 | 1.07 – 1.18 | 1.32 – 1.43 | 1.32 – 1.43 | 0.77 – 1.06 |
| Highly  congruent (HC) | 1.13 – 1.33 | 1.29 – 1.41 | 1.59 – 1.71 | 1.59 – 1.71 | 0.77 – 1.06 |
| Moderately incongruent (MI) | 1.13 – 1.33 | 0.70 – 0.76 | 0.85 – 0.93 | 0.85 – 0.93 | 0.77 – 1.06 |
| Highly incongruent (HI) | 1.13 – 1.33 | 0.58 – 0.63 | 0.71 – 0.78 | 0.71 – 0.78 | 0.77 – 1.06 |
