# Supplementary material for: Visuo-spatial (but not verbal) executive working memory capacity modulates susceptibility to non-numerical visual magnitudes during numerosity comparison
Source: PLoS One. 2019 Mar 27;14(3):e0214270. doi: 10.1371/journal.pone.0214270 (PMC6436736; doi:10.1371/journal.pone.0214270)
Supplement: S3 Table — (DOCX) [file pone.0214270.s003.docx]

**S3 Table. The list of trial-based ANCOVAs on performance using Group as the within-trial factor, Condition as the between-trial factor along with covariates.**

| **Within-trial factor** | **Between-trial factor** | **Covariate** |
| --- | --- | --- |
| Visuo-spatial EWM group (High/ Low) | Condition  (Congruent/ Incongruent) | Numerosity ratio, Inter dot space ratio, RT |
| Visuo-spatial EWM group (High/ Low) | Condition  (Congruent/ Incongruent) | Size ratio, Inter dot space ratio, RT |
| Verbal EWM group (High/ Low) | Condition  (Congruent/ Incongruent) | Numerosity ratio, Inter dot space ratio, RT |
| Verbal EWM group (High/ Low) | Condition  (Congruent/ Incongruent) | Size ratio, Inter dot space ratio, RT |
