# Supplementary material for: Visuo-spatial (but not verbal) executive working memory capacity modulates susceptibility to non-numerical visual magnitudes during numerosity comparison
Source: PLoS One. 2019 Mar 27;14(3):e0214270. doi: 10.1371/journal.pone.0214270 (PMC6436736; doi:10.1371/journal.pone.0214270)
Supplement: S4 Table — (DOCX) [file pone.0214270.s004.docx]

**S4 Table. The list of trial-based hierarchical regression analyses conducted on accuracy for each group.**

| **Group** | **Dependent variable** | **Predictors** |
| --- | --- | --- |
| High visuo-spatial EWM | Mean accuracy | Numerosity ratio, Size ratio, Inter dot space ratio, RT |
| Low visuo-spatial EWM | Mean accuracy | Numerosity ratio, Size ratio, Inter dot space ratio, RT |
| High verbal EWM | Mean accuracy | Numerosity ratio, Size ratio, Inter dot space ratio, RT |
| Low verbal EWM | Mean accuracy | Numerosity ratio, Size ratio, Inter dot space ratio, RT |
