# Supplementary figures and images for: Visuo-spatial (but not verbal) executive working memory capacity modulates susceptibility to non-numerical visual magnitudes during numerosity comparison
Source: PLoS One. 2019 Mar 27;14(3):e0214270. doi: 10.1371/journal.pone.0214270 (PMC6436736; doi:10.1371/journal.pone.0214270)

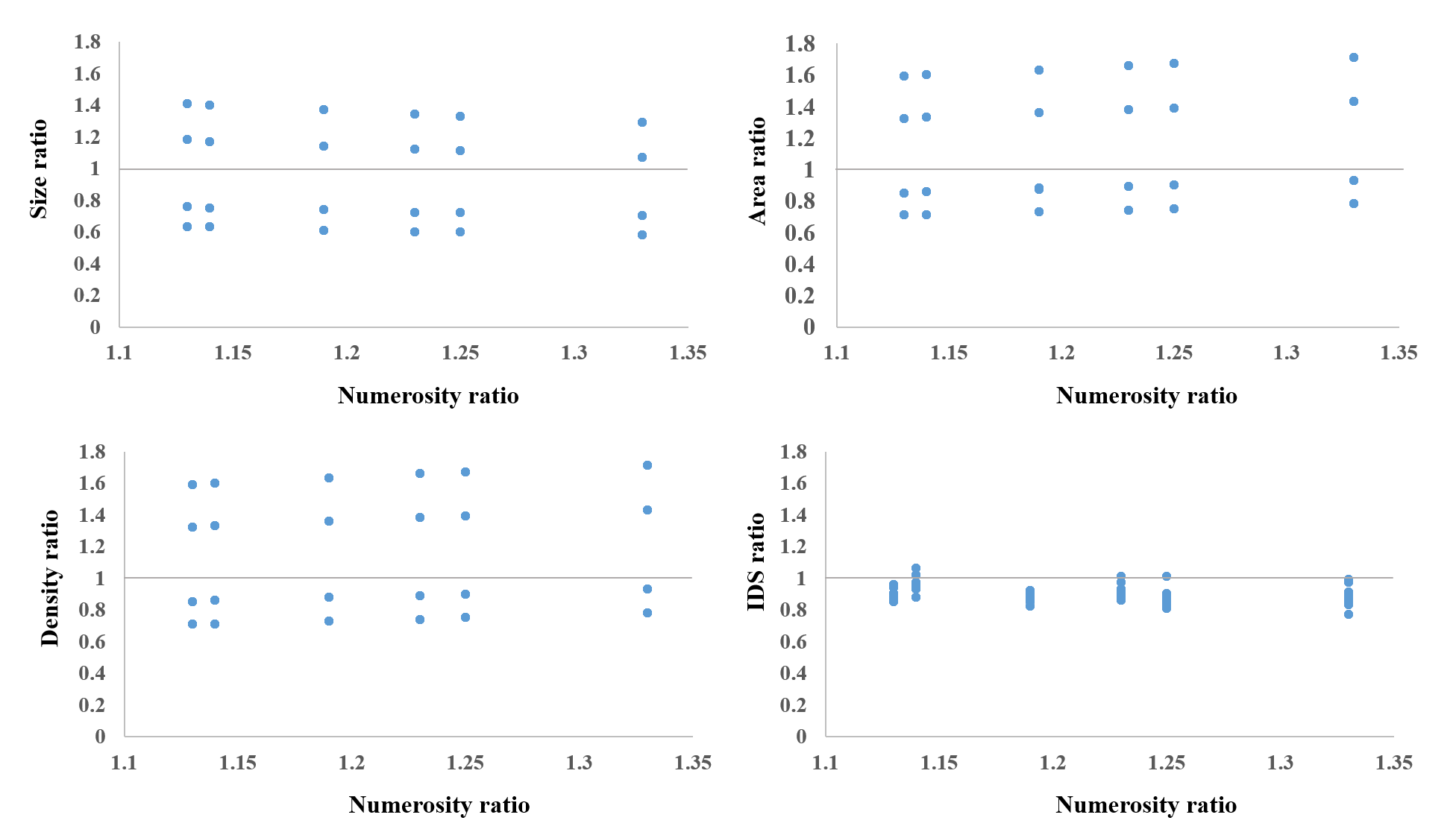

Supplement: S1 Fig — Trial types are depicted in four different ways, in terms of the relationships between the numerosity ratio and each non-numerical visual magnitude ratio. Each point represents a certain type of trial (contains 10 trials each). The horizontal line in each figure defines the boundary that separates the Congruent (> 1) and Incongruent (< 1) conditions. (TIF) [file pone.0214270.s005.tif]

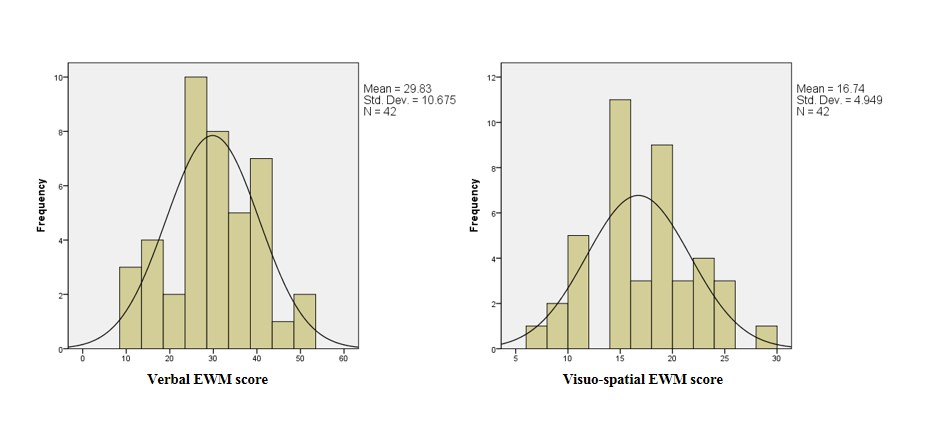

Supplement: S2 Fig — The analysis of skewness and kurtosis revealed that none of the scores exceeded + or – 1.00. (TIF) [file pone.0214270.s006.tif]
