# Supplementary material for: Visuo-spatial (but not verbal) executive working memory capacity modulates susceptibility to non-numerical visual magnitudes during numerosity comparison
Source: PLoS One. 2019 Mar 27;14(3):e0214270. doi: 10.1371/journal.pone.0214270 (PMC6436736; doi:10.1371/journal.pone.0214270)
Supplement: S1 Appendix — Here we provide additional descriptions about experimental tasks, analysis methods and behavioral performance on operation and rotation span tasks. (DOCX) [file pone.0214270.s007.docx]

**Supplementary methods**

**Participants**

All participants were native Korean speakers and received formal mathematics education in South Korea.

**Tasks and materials**

**Numerosity comparison task**

Each dot array contained black dots within a white rectangular background (580 x 434 pixels). The diameter of the dots ranged from 3.97 to 7.75 mm. E-prime software was used for stimulus presentation and data collection (Psychology Software Tools, Inc., 2002).

Trials were evenly divided into four conditions (Moderately congruent, Highly congruent, Moderately incongruent, Highly congruent) based on the degree of correlation between the ratios of numerosity and non-numerical visual magnitudes (dot size, cumulative dot area and dot density which are all inherently related with one another) (S1 Fig, S2 Table). All ratios of visual magnitudes were calculated by dividing the magnitude of the more numerous array by that of the less numerous array (e.g., the numerosity ratio of a pair of dot arrays with set sizes of 6:5 would be 1.2).

**Operation span task (Verbal EWM) stimulus dimensions**

On each trial, a math problem was presented with numbers and characters in black, Arial bold type, font size 24. After solving the math problem, a letter of the alphabet was presented in black, Arial bold type, font size 28.

**Rotation span task (Visuo-spatial EWM) stimulus dimensions**

Black arrows were presented on a white rectangular background (200 x 200 pixels) with 2 different lengths (18mm, 55mm). The stimulus dimensions were the same as in Foster et al. (2015). The Korean letter (e.g., “화”) was presented in black, bold font, size 200.

**Methods of analyses**

The trial-based analyses were conducted by an ANCOVA (S3 Table) and hierarchical regression analysis (S4 Table) on the average performance of each trial using the ratio of visual magnitudes (numerical and non-numerical visual magnitudes) as covariates (in the ANCOVA) or predictors (in the regression analysis) for each group separately (note, in this trial-based analysis, it is not possible to use EWM scores as a continuous, between-subject variable; see S1 Table).

**Supplementary Results**

**Behavioral performance on operation and rotation span tasks**

The mean scores of the Operation Span (verbal EWM) and Rotation Span (visuo-spatial EWM) tasks were 29.83 (SD = 10.68) and 16.74 (SD = 4.95), respectively. The distributions of EWM scores are shown in S2 Fig.
